# Single heartbeat ECG authentication: a 1D-CNN framework for robust and efficient human identification

**DOI:** 10.3389/fbioe.2024.1398888

**Published:** 2024-07-04

**Authors:** Ana Rahma Yuniarti, Syamsul Rizal, Ki Moo Lim

**Affiliations:** ^1^ Department of IT Convergence Engineering, Kumoh National Institute of Technology, Gumi-si, Republic of Korea; ^2^ Department of Computer Engineering, Universitas Pendidikan Indonesia, Bandung, Indonesia; ^3^ School of Electronics and Electrical Engineering, Telkom University, Bandung, Indonesia; ^4^ Department of Medical IT Convergence Engineering, Kumoh National Institute of Technology, Gumi-si, Republic of Korea; ^5^ Meta Heart Inc., Gumi-si, Republic of Korea

**Keywords:** electrocardiogram (ECG), authentication, biometrics, 1D-CNN, convolutional neural network, SMOTE, single heartbeat, identification

## Abstract

This study proposes a small one-dimensional convolutional neural network (1D-CNN) framework for individual authentication, considering the hypothesis that a single heartbeat as input is sufficient to create a robust system. A short segment between R to R of electrocardiogram (ECG) signals was chosen to generate single heartbeat samples by enforcing a rigid length thresholding procedure combined with an interpolation technique. Additionally, we explored the benefits of the synthetic minority oversampling technique (SMOTE) to tackle the imbalance in sample distribution among individuals. The proposed framework was evaluated individually and in a mixture of four public databases: MIT-BIH Normal Sinus Rhythm (NSRDB), MIT-BIH Arrhythmia (MIT-ARR), ECG-ID, and MIMIC-III which are available in the Physionet repository. The proposed framework demonstrated excellent performance, achieving a perfect score (100%) across all metrics (i.e., accuracy, precision, sensitivity, and F1-score) on individual NSRDB and MIT-ARR databases. Meanwhile, the performance remained high, reaching more than 99.6% on mixed datasets that contain larger populations and more diverse conditions. The impressive performance demonstrated in both small and large subject groups emphasizes the model’s scalability and potential for widespread implementation, particularly in security contexts where timely authentication is crucial. For future research, we need to examine the incorporation of multimodal biometric systems and extend the applicability of the framework to real-time environments and larger populations.

## 1 Introduction

Advancements in technology have significantly transformed various sectors, including banking and personalized services, by shifting the majority of them to the digital realm. Consequently, ensuring the security of data and applications has become a pressing concern, given that services are gradually easier to access (more open) and are simultaneously more prone to malicious third-party attacks (Shahim, 2021). Security systems that depend on external components such as tokens, passwords, or identification (ID) cards are acknowledged for their robust security when correctly used but are also susceptible to theft, loss, and replication Shaheed et al. (2021). As a result, biometric authentication has been considered an important means of digital security, providing secure authentication by identifying individuals based on their unique biological (anatomical and physiological) and behavioral features (Nguyen et al., 2018; Hammad et al., 2019a).

**FIGURE 1:**
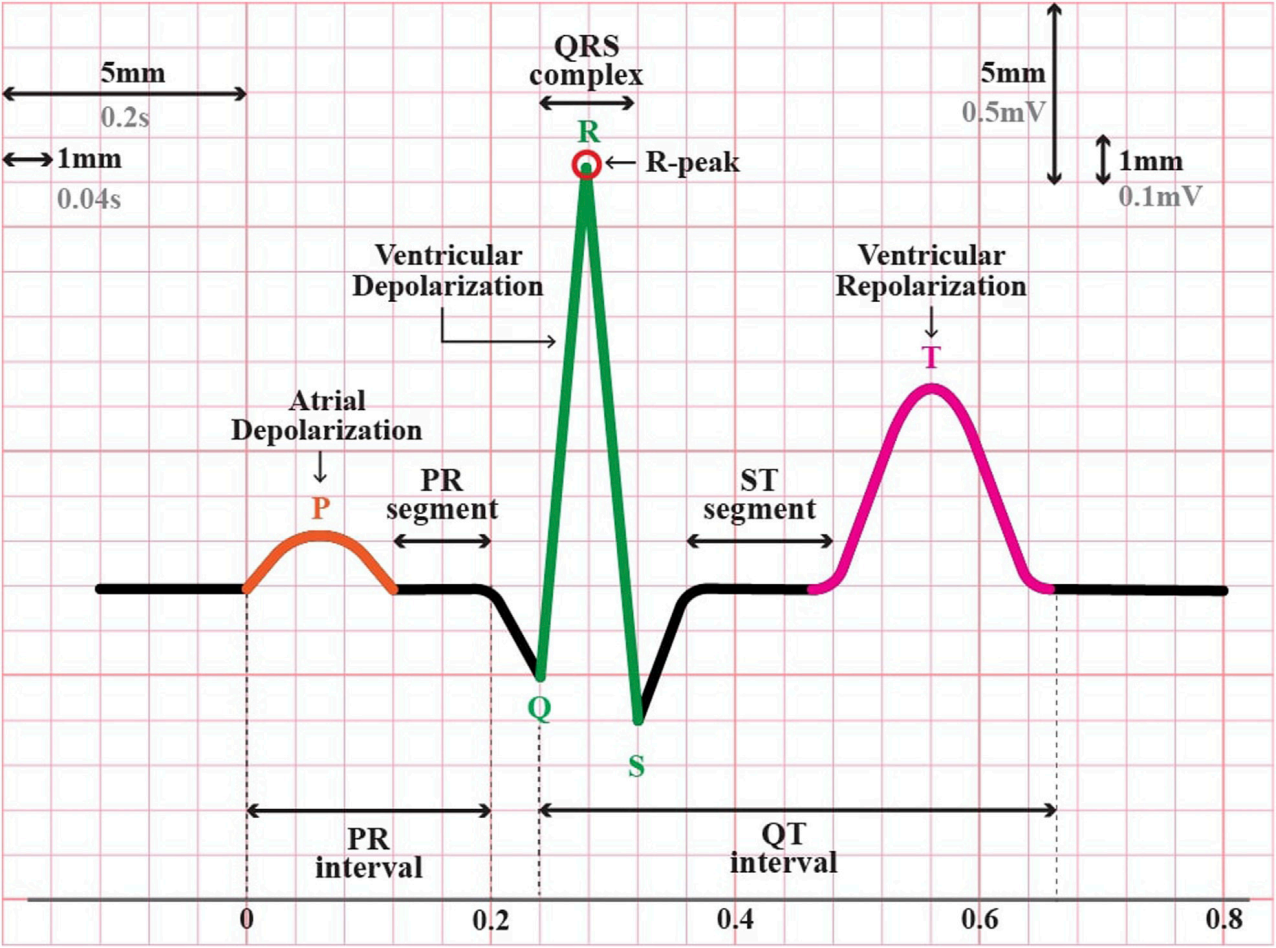
The typical electrocardiogram (ECG) waveform in one cycle of a heartbeat.

Personal identification through different biometric identifiers, such as fingerprints, voice, gait, and iris, has been implemented worldwide on a large scale (AlDuwaile and Islam, 2021). However, the rapid advancement of falsification technology has created vulnerability to attacks on these biometric traits (Patro et al., 2019). For instance, fingerprints can be recreated using latex (An et al., 2020), voice can be manipulated with advanced recording tools (Hamdan and Mokhtar, 2018), the face can be counterfeited by artificial masks (Erdogmus and Marcel, 2014; Hamdan and Mokhtar, 2018), and the iris can be falsified by using customized contact lenses or artificial eyes (Wang et al., 2007; Nguyen et al., 2018; Kauba et al., 2020). Given these circumstances, in recent years, there has been a growing interest among researchers in exploring the feasibility of utilizing “hidden” traits that encompass the element of “aliveness,” such as Electroencephalogram (EEG) or brain signals, and Electrocardiogram (ECG) or heart signals (Chiu et al., 2021). In terms of signal acquisition, ECG is notably simpler than EEG (Pinto and Cardoso, 2019; AlDuwaile and Islam, 2021). For instance, ECG signals can be easily obtained from fingers (Islam and Alajlan, 2017; Gwynn et al., 2021), whereas EEG signals typically require more sophisticated equipment and a more complex setup (Agarwal et al., 2020; AlDuwaile and Islam, 2021). Additionally, the ECG waveform is relatively straightforward, strong, and focused, resulting in a distinct and clear pattern compared to EEG. In a single heartbeat or one cycle of cardiac activity, a typical ECG waveform consists of three major segments: P, QRS, and T waves (see Figure 1). This pattern was repeated and was relatively consistent over time. Therefore, utilizing ECG signals as a new biometric modality could be more acceptable for real-world applications.

Other key strengths of ECG utilization for biometric authentication are as follows. First, ECGs have been proven to be unique among individuals due to variations in both physiological and geometrical characteristics of the heart among different individuals (inter-individual variability) (AlDuwaile and Islam, 2021; Yuniarti and Rizal, 2022). The pattern of the ECG waveform on each individual is reliant on morphological aspects, such as the thickness of the cardiac muscle, shape, and size of the heart (El Boujnouni et al., 2022). Second, ECG recordings are exclusive to living entities and can be used to verify the authenticity of access attempts, distinguishing between human and non-human sources (Pinto and Cardoso, 2019; Rathore et al., 2020). On the other hand, adding a “liveness detection” feature to certain biometrics other than ECG is computationally costly and falls short of system security. Third, the physiological data in ECG have been used in various clinical applications, including cardiac disease diagnosis, emotion recognition, and psychophysiological state assessment (Goshvarpour and Goshvarpour, 2019). This, in turn, paves the way for new cutting-edge research opportunities to harness ECGs in the creation of fusion applications serving dual purposes: health monitoring and biometrics (Tan and Perkowski, 2017; Zhang et al., 2017; Cosoli et al., 2021).

Over the last decade, considerable effort has been made to develop various algorithms using ECG for individual authentication (Hammad et al., 2019b; Donida Labati et al., 2019; Pinto and Cardoso, 2019; Ihsanto et al., 2020; Srivastva et al., 2021). The pioneering research by Biel et al. (2001) portrayed the viability of principal component analysis (PCA) along with soft independent modeling of class analogy (SIMCA) classifiers to identify 20 individuals based on ten fiducial features extracted from 12-lead ECG signals. Lee et al. (2018) introduced a curvature-based vertex selection technique using polygonal approximation to reduce the number of samples required for detecting fiducial points of the ECG signal, specifically the QRS complex. The effectiveness of the algorithm was verified through experiments using the QT-DB and MIT-ARR databases. In recent years, researchers have explored the use of DL-based approaches such as CNN. Pinto and Cardoso (2019) applied transfer learning of 1D-CNN with Euclidean distance to classify 290 individuals in the Physikalisch-Technische Bundesanstalt (PTB) database (Bousseljot et al., 2004). The input for the network was multiple heartbeats that were segmented blindly every 5 s. However, its accuracy was stuck at 91%. Hong et al. (2019) employed a 2D-CNN-based algorithm, that is, the Inceptionv3 model, and transferred learning to identify 200 subjects in the PTB database. They converted the ECG signals into an image using spatial correlation-based and temporal correlation-based signals as input to the network and achieved 98% accuracy. Donida Labati et al. (2019) conducted an experiment using the same database, PTB, but only 52 healthy subjects were employed and achieved a perfect accuracy of 100%. They used a 2D-CNN architecture to extract a set of feature vectors composed of multiple QRS complexes from ECG samples. Later work by Ihsanto et al. (2020) also reported a perfect accuracy of 100% with a residual depthwise separable CNN, but using different databases, that is, ECG-ID and MIT-ARR. For the ECG-ID database, 100% accuracy was obtained with a minimum of eight heartbeats, and for the MIT-BIH database, a minimum of six heartbeats was required. Li et al. (2020) introduced a cascaded CNN consisting of an F-CNN to extract heartbeat features and an M-CNN for template comparison. They used five different databases, including FANTASIA, CEBSDB, NSRDB, STDB, and AFDB, for the experiments and attained identification rates of 99.3%, 93.1%, 91.4%, 92.7%, and 89.7%, respectively, using a single heartbeat input. They also explored the performance of the cascaded CNN for several heartbeats (3, 5, 8, 10, 15, and 20 beats). However, they recommended using at least three heartbeats for efficient performance with identification rates of 99.9%, 95.0%, 96.1%, 95.2%, and 90.9% for FANTASIA, CEBSDB, NSRDB, STDB, and AFDB, respectively. Srivastva et al. (2021) proposed PlexNet that leverages the benefits of both transfer learning and ensemble learning of ResNet and DenseNet based on three sequential heartbeats extracted from ECG signals. They attained a 99.66% accuracy with multi-session datasets using PTB and CYBHi. The majority of previous studies require a lengthy segment of the ECG signal consisting of multiple heartbeats to achieve optimal accuracy. This presents practical challenges for real-world applications, particularly when capturing and processing time are costly.

AlDuwaile and Islam (2021) investigated the effectiveness of employing a single heartbeat for human biometric recognition. They employed four pre-trained models (GoogleNet, ResNet, MobileNet, and EfficientNet) along with CNN to assess the impact of different types of segments of ECG signals, including R-centered segments (a fixed-length segment around the R-peak), R-R segment, and P-P segment. These segments must be converted into continuous wavelet transformation (CWT) images before being fed into the DL network. They reported an accuracy of 99.90% with the PTB dataset containing a single-session ECG recording. However, an accuracy between 94.18% and 98.20% was achieved with ECG-ID datasets under multi-session and mixed-session, respectively. Prakash et al. (2023) also investigated the potential application of a short segment of ECG signals for biometric recognition using a Siamese network (twin network). The short segment around R-peak was generated by clipping a series of ECG signals into single, dual, and triple heartbeats, and converting them into binary images. The Siamese network was constructed using a pair of identical 1D-CNN backbones that shared the same parameters and weights. It takes two images as inputs and returns a similarity (distance) metric that indicates how similar they are. They achieved accuracies of 91%, 99.85%, and 99.90% using single, dual, and triple ECG beats, respectively, when 90 individuals were identified in an ECG-ID database.

Research with a single-heartbeat paradigm mostly requires the transformation of ECG signals (1D) into an image (2D). This work explores the feasibility of a single heartbeat utility without the need to transform it into a 2D space using 1D-CNN. Moreover, the 1D-CNN framework was also constructed using only two layers of convolutional networks to simplify the complexities of the architecture. This study targets robust authentication accuracy across diverse conditions (small and large subject populations, normal and abnormal hearts, imbalanced and balanced samples, or single and mixed sessions). We achieved this by assessing the proposed framework on various databases embodying such conditions from a public repository (Physionet.org) and comparing the outcomes with those of other approaches reported in the literature. These databases are the MIT-Normal Sinus Rhythm Database (NSRDB) (The Beth Israel Deaconess Medical Center, 1990), MIT-BIH Arrhythmia (MIT-ARR) (Moody and Mark, 1992), ECG-ID (Lugovaya, 2011), and MIMIC-III waveform databases (Moody et al., 2020). The assessment was performed individually and in a mixture of all those databases. The main contributions of our paper are as follows.

1. Proposing a 1D convolutional neural network (1D-CNN) framework for individual authentication using a single heartbeat extracted from the ECG signal.
2. Utilizing the segment between two consecutive R-peaks (R-R segment) to represent a single heartbeat, capturing the complete systolic and diastolic phases of the cardiac cycle.
3. Employing a rigid length thresholding procedure combined with interpolation to generate consistent single heartbeat samples across subjects.
4. Exploring the benefits of the synthetic minority oversampling technique (SMOTE) to address imbalanced sample distributions among individuals.
5. Comprehensive evaluation of the proposed framework on individual and mixed datasets from public ECG databases (NSRDB, MIT-ARR, MIMIC-III, and ECG-ID), demonstrating high authentication accuracy across diverse conditions.
6. Achieving perfect authentication scores (100% accuracy, precision, sensitivity, and F1-score) on individual NSRDB and MIT-ARR datasets with balanced sample distributions.
7. Demonstrating the scalability and potential for widespread implementation of the proposed framework, particularly where timely authentication is crucial.
8. Comparative analysis with state-of-the-art methods, highlighting the superior performance and computational efficiency of the proposed 1D-CNN approach for single heartbeat ECG authentication.

## 2 Materials and methods

### 2.1 Problem definition

Authentication problems using ECG signals can be differentiated into two tasks that serve different purposes: verification (binary problem) or identification (multi-class problem). In the verification task, the system compares the individual’s ECG signals against the pre-enrolled data (templates) to determine whether the presented identity matches or corresponds to the claimed identity. Thus, the output of the verification system is binary–either a match (verification success) or no match (verification failure). Meanwhile, in the identification task, the system compares the presented ECG signals of a subject against templates from all subjects in the database and find the best match to determine the subject’s identity. Our work is focused on identification rather than verification, where the goal is to correctly classify each subject into one of several possible subjects. Specifically, our study was performed within a closed-set environment, meaning that no new individuals can be recognized outside of the predefined group in the database. The training and testing sets share the same set of classes, and every instance in the test set belongs to one of the known classes observed during training.

### 2.2 Database overview

In this study, four databases obtained from the well-known freely accessible database Physionet were used to analyze and evaluate the efficacy of the proposed framework. These four databases are NSRDB (The Beth Israel Deaconess Medical Center, 1990), MIT-BIH Arrhythmia (Moody and Mark, 1992), ECG-ID (Lugovaya, 2011), and MIMIC-III (Moody et al., 2020), which contain normal and abnormal heart conditions or either single or multi-session recording. In this study, we utilized ECG signals from 18 subjects in the NSRDB, 27 subjects in the MIT-BIH, 90 subjects in the ECG-ID, and 83 subjects in the MIMIC-III databases. The experiments were performed individually on each database and in combination with the databases. The MIXED-1 dataset was obtained from the combined ECG-ID (90 subjects) and MIMIC-III (83 subjects) data, which produced 173 subjects. MIXED-2 is a combination of three databases (ECG-ID, MIMIC-III, and MIT-BIH) that generated 200 subjects. The last combination, MIXED-3, derived from the ECG-ID, MIMIC-III, MIT-BIH, and NSRDB databases, resulted in 218 subjects. A summary of the datasets used in this study is presented in Table 1.

1. NSRDB: This database contains continuous ECG recordings for approximately 24 h from 18 subjects (five men aged 26–45 years and 13 women aged 20–50 years). The ECG signals included in this database showed no notable arrhythmic conditions. The recordings were digitized at a sampling rate of 128 Hz and subsequently represented as 12-bit binary sequences. In this database, there are two types of ECG signals, namely, “ECG1” and “ECG2” which are taken from different leads on a subject’s body. For our experiment, we used the “ECG1″ signals.
2. MIT-ARR: This database includes 48 half-hour ECG recordings from 47 subjects (25 men aged between 32 and 89 years and 22 women aged from 23 to 89 years who were associated with different clinical pathologies. Each record contains two ECG channels sampled at 360 Hz with an 11-bit resolution over a 10 mV range. In this study, we only used recordings from 27 subjects with channel “MLII”.
3. MIMIC-III: This database comprises long-term quasi-continuous recordings of ECG signals along with up to eight other physiological waveforms (Photoplethysmogram or PPG, Arterial Blood Pressure or ABP, respiration, *etc.*) from approximately 30,000 ICU patients. The signal duration in this database varied depending on the duration of each patient’s stay in the ICU. The recorded signals were digitized at a sampling rate of 125 Hz with 8-, 10-, or (occasionally) 12-bit resolutions.
4. ECG-ID: This database comprises 310 ECG recordings collected from 90 subjects (44 males and 46 females aged 13–75 years) for approximately 20 s. The number of ECG records collected from each subject varied, ranging from two recordings acquired within a single day to as many as 20 recordings collected periodically over a period of 6 months. Each ECG record encompasses Lead-I data, which are digitized at a sampling rate of 500 Hz with a 12-bit resolution over a nominal range of 10 mV. There are two types of ECG signals provided in this database, namely, “ECG I” (unfiltered signals) and “ECG I Filtered”. In this study, an unfiltered signals was used.
5. MIXED-1: This dataset was created by combining two existing datasets, that is the ECG-ID and MIMIC-III datasets, resulting in a total of 173 subject datasets.
6. MIXED-2: This dataset was created by combining three existing datasets, that is ECG-ID, MIMIC-III, and MIT-BIH Arrhythmia datasets, resulting in 200 subject datasets.
7. MIXED-3: This dataset was created by combining four existing datasets, that is ECG-ID, MIMIC-III, MIT-BIH Arrhythmia, and NSRDB datasets, resulting in a total of 218 subject datasets.

**TABLE 1:** The summary of the datasets adopted in our experiments.

No.	Database	Sampling rate (Hz)	# Samples per subject	Session	Condition
1	NSRDB	128	18	single	healthy
2	MIT-ARR	360	27	single	arrhythmia (along with 18 other diseases)
3	MIMIC-III	125	83	single	healthy and arrhythmia
4	ECG-ID	500	90	mix	healthy
5	MIXED-1^a^	250	173	mix	healthy and arrhythmia
6	MIXED-2^b^	250	200	mix	healthy and arrhythmia
7	MIXED-3^c^	250	218	mix	healthy and arrhythmia

^a^ MIXED-1: ECGID + MIMIC III.

^b^ MIXED-2: ECGID + MIMIC III + MIT-ARR.

^c^ MIXED-3: ECGID + MIMIC III + MIT-ARR + NSRDB.

### 2.3 Proposed framework

**FIGURE 2:**
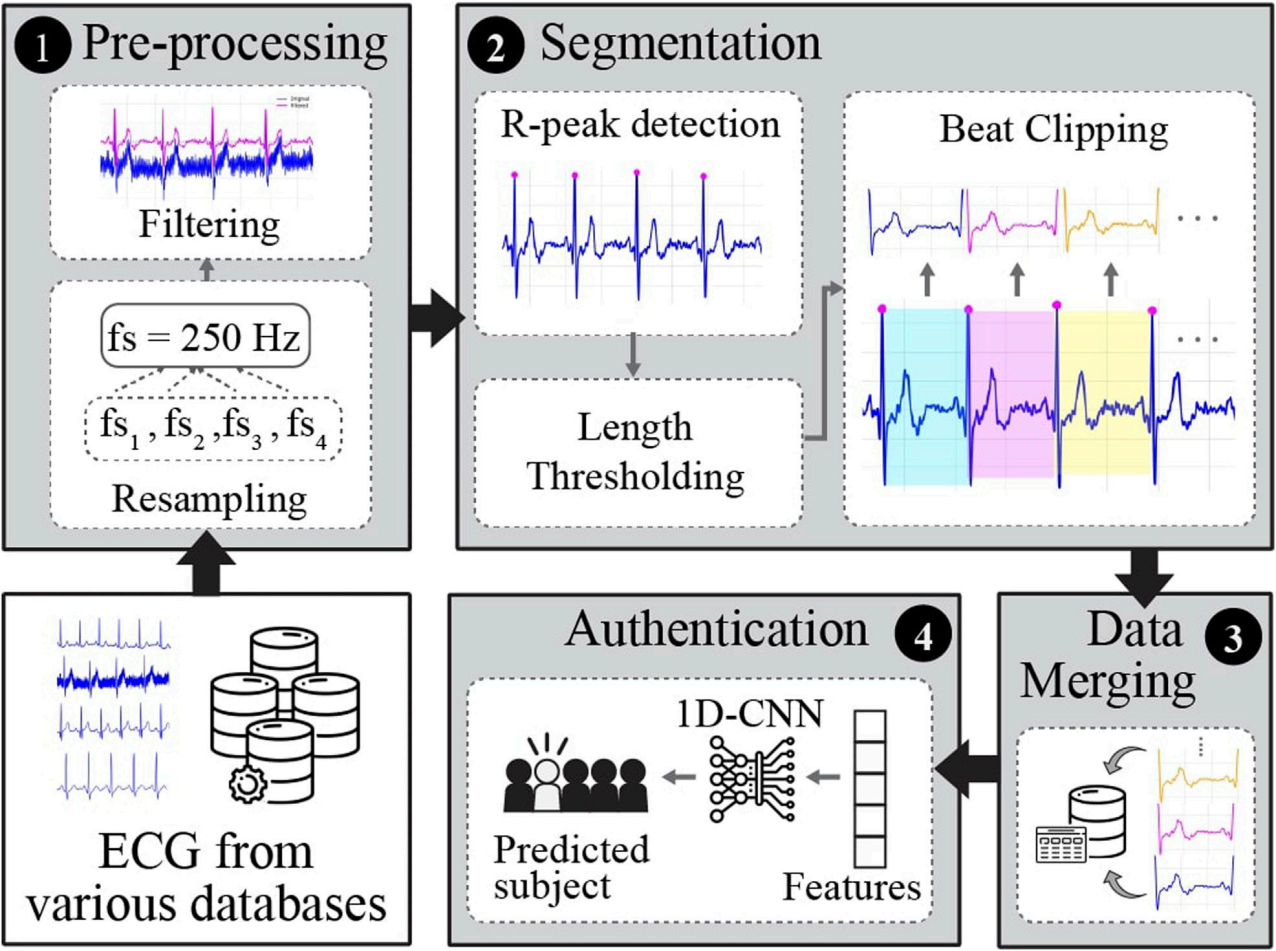
The general pipeline of the proposed framework for single heartbeat ECG-based identification.

The overall pipeline of the proposed framework for ECG-based biometric authentication using a 1D-CNN is illustrated in Figure 2. It encompasses four stages: pre-processing, segmentation, data merging, and authentication.

#### 2.3.1 Pre-processing

This stage aims to enhance the quality of raw ECG recordings and prepare them for further experiments. In this study, pre-processing included frequency resampling and filtering. ECG signals from various databases were recorded at different sampling frequencies. Hence, the signals were first resampled to 250 Hz to set the uniformity and standardization of all the databases utilized in this study. The majority of raw ECG signals from various databases are contaminated with noise, such as power-line interference, baseline wander (breathing or body movement), or muscle (electromyographic) noise. To attenuate the noise from raw ECG signals, we applied a 3rd-order Butterworth bandpass filter (BPF) with a low cutoff frequency of 0.6 Hz and a high cutoff frequency of 40 Hz.

#### 2.3.2 Segmentation

In this stage, the continuously filtered ECG signals were segmented into individual heartbeats. There are two types of ECG segmentation methods: fiducial and non-fiducial. The Fiducial method relies on specific points in the ECG waveform for segmentation, such as the R-peak and P-wave onset or offset, Q-wave onset, QRS onset or offset, and T-wave onset or offset (Figure 1). In contrast, the non-fiducial method does not consider specific points. Instead, it analyzes the entire signal using more complex algorithms such as transforming the ECG signal into the frequency domain using Fourier transform, decomposing the ECG signals into different frequency components using Wavelet Transform, application of a sliding window technique, template matching, or machine learning techniques (Menon et al., 2022; Haleem and Pecchia, 2022. In this study, a fiducial approach was adopted.

Most studies in the literature utilize the R-centered paradigm (i.e., a short segment around the R-peak) to clip the ECG signal into a single heartbeat. This is performed by detecting the location of R-peak, which is the most notable feature of ECG waveform. Then, they approximated the segment from the detected R-peak to its left and right (AlDuwaile and Islam, 2021; Prakash et al., 2023). While this approach can capture the complete P-QRS-T complex when implemented correctly, it may not accurately represent the entire cardiac cycle for individuals with varying heart rates or heart rate variability within the same subject. The variability in heart rates can affect the length and features of the P-QRS-T complex. Hence, in this study, to represent a single heartbeat that encapsulates the complete systolic and diastolic phases, we chose a segment between two successive R-peaks (R-R segment). This approach ensures the inclusion of all phases of the cardiac cycle regardless of heart rate variability and provides a more comprehensive representation of each heartbeat.

**FIGURE 3:**
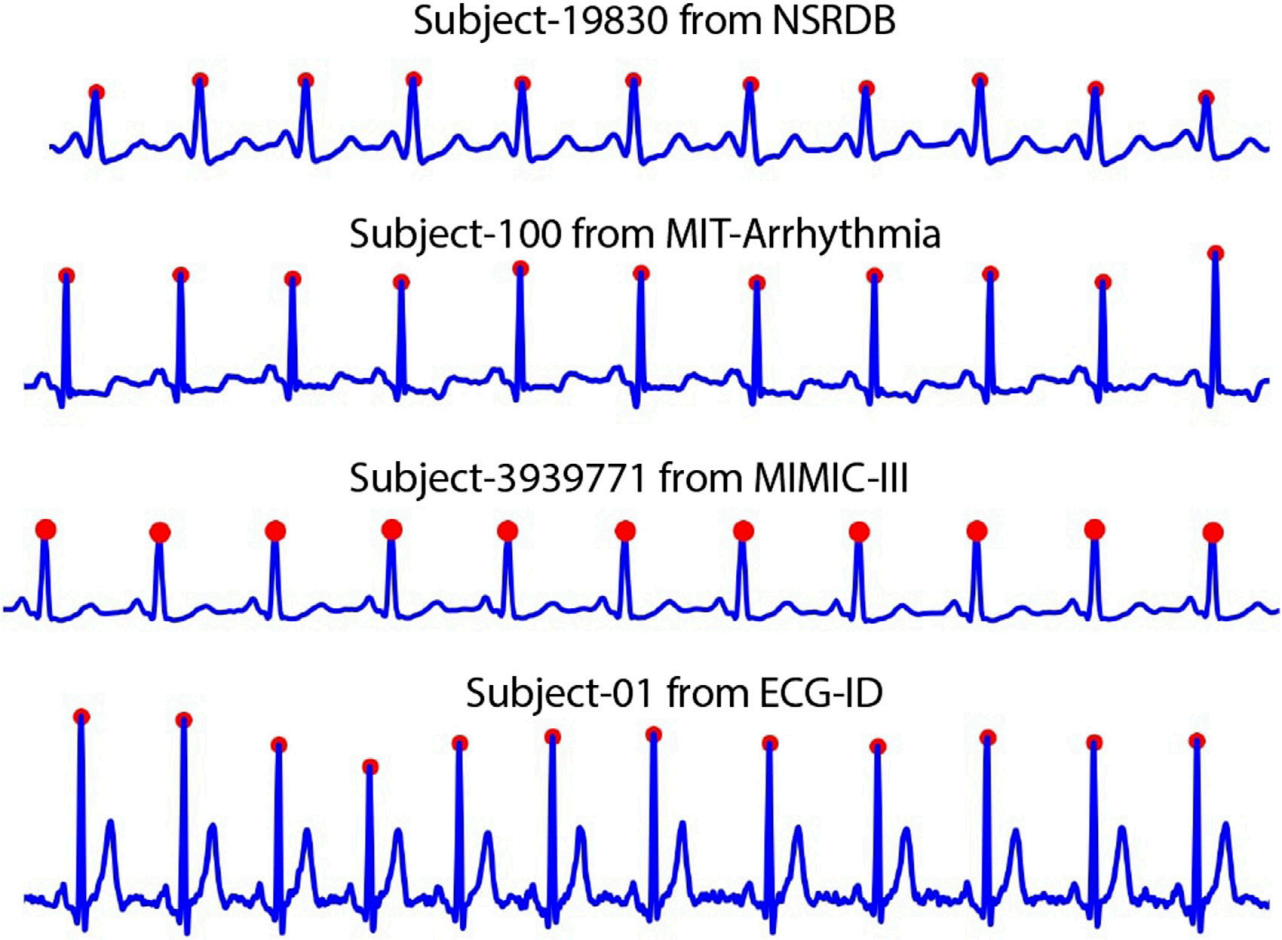
The results of R-peak detection algorithm in various databases.

**FIGURE 4:**
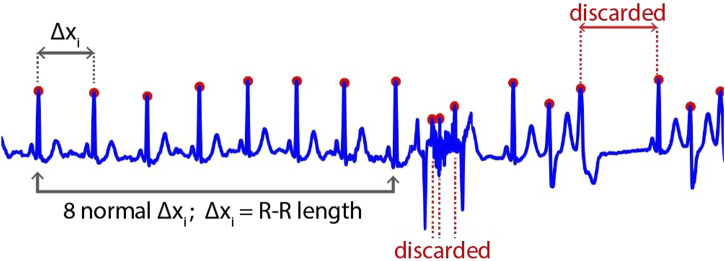
The inappropriate result of R-peak detection algorithm.

First, the location of the R-peak is detected by applying the Pan and Tompkins (1985), which is used in common QRS detection. The results of this process are shown in Figure 3. However, in some cases, the R-peak is not properly detected, as shown in Figure 4. Thus, to discard unwanted components in such cases, we applied rigid length thresholding after detecting the R-peak. This is performed by initially calculating the length of two consecutive detected R peaks using the following equation:

$$\Delta x_{i}=x_{i}-x_{i-1};i=1,\ldots ,N-1$$ (1)

where $\Delta x_{i}$ is the length of the two consecutive detected R-peaks. We then took only eight RR-lengths within the standard or normal range. The normal range is assumed if, between two R peaks, there are no fluctuating signals (artifact-free), no T-peak is detected, or no R-peak is skipped, based on our manual observation. We then calculated the mean (2) and standard deviation (3) of the normal R-R length using the following equations:

$$\mu =\frac{1}{N}\overset{N}{\underset{i=1}{\sum }}\Delta x_{i};N=8$$ (2)

$$\sigma =\sqrt{\frac{1}{N}\overset{N}{\underset{i=1}{\sum }}{\left(\Delta x_{i}-\mu \right)}^{2}};N=8$$ (3)

**FIGURE 5:**
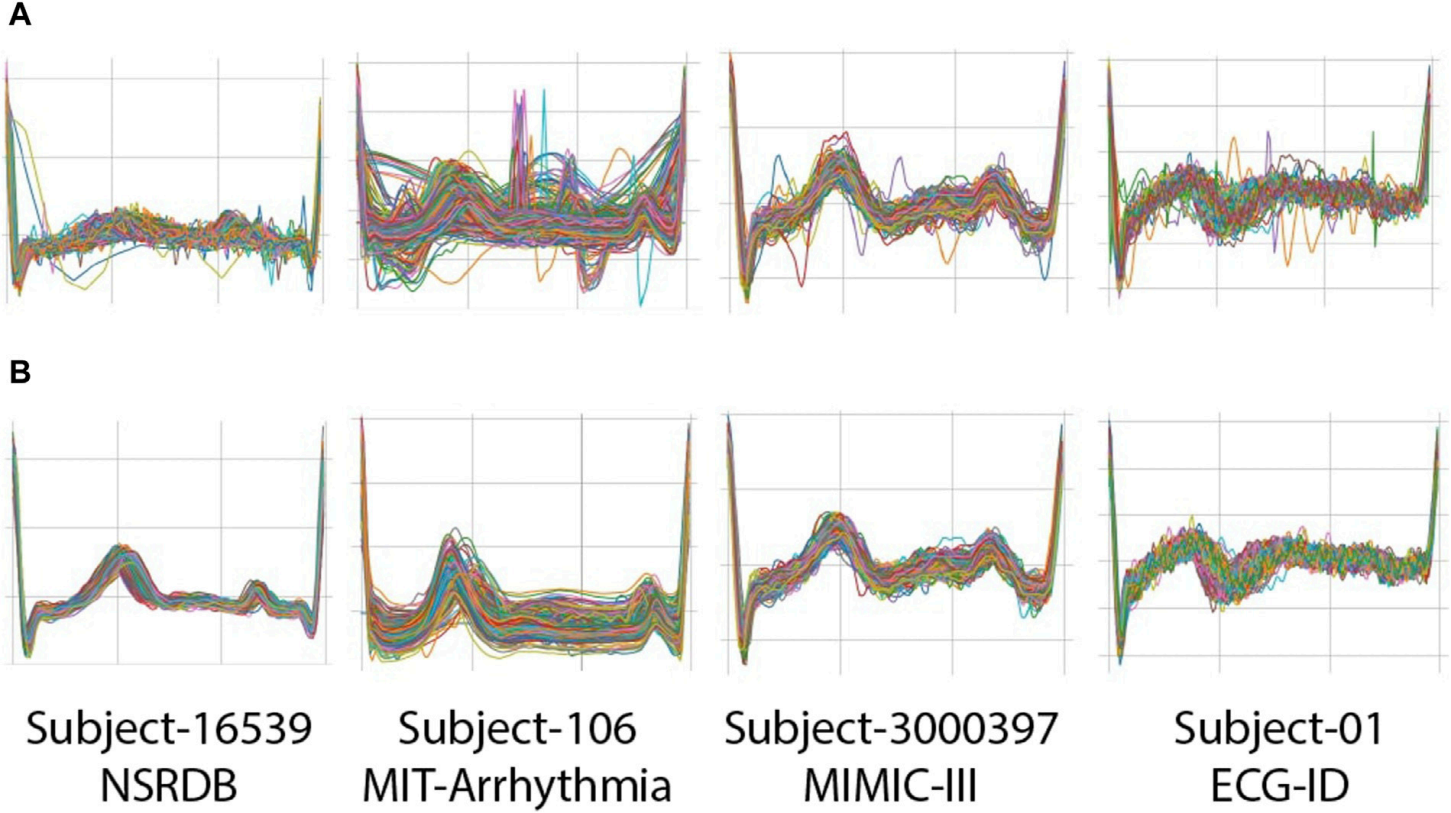
Examples of clipped beats (R-R segments) from four subjects on NSRDB, MIT-ARR, MIMIC-III, and ECG-ID databases: **(A)** before applying rigid thresholding; **(B)** after applying rigid thresholding.

Then, two thresholds are set to discard the unwanted signals:

$$th_{1,2}=\mu \pm \sigma$$ (4)

where $th_{1,2}$ are the lower and upper thresholds, respectively. The R-R length that is less than the lower threshold $\left(th_{1}\right)$ or higher than the upper threshold $\left(th_{2}\right)$ is discarded. The selected signals were then interpolated to the reference length of 150 samples to set the uniformity of the R-R length, representing a single heartbeat for each subject. Figure 5 shows the example results of the clipped beats from two different subjects with and without the application of rigid thresholding in our study.

#### 2.3.3 Data merging

The end of the segmentation stage produces several clipped beats corresponding to each subject. All clipped beats from all subjects were then merged into one CSV file for further analysis and experiments using the deep-learning model. We first prepared 4 CSV files corresponding to each database, that is, NSRDB, MIT-ARR, MIMIC-III, and ECG-ID. Next, we created three other datasets (MIXED-1, MIXED-2, MIXED-3) using the combination of them.

#### 2.3.4 Deep-learning training

Initially, we experimented with several architectures, including models with three or more convolutional layers. However, these models exhibited overfitting and did not achieve the desired accuracy, primarily due to their increased complexity, which was not well-suited to our dataset. The number of convolutional layers was reduced to two after empirical testing. This simplification helped mitigate overfitting and improved the generalization capability of the model. We experimented with various filter sizes ranging from small (3, 5) to larger filters (7, 9). Smaller filters provided better feature extraction in our context, capturing the essential characteristics of the ECG signal. Different numbers of kernels (16, 32, 64) were tested. We found that starting with 16 kernels in the first layer and increasing to 32 in the second layer achieve a good balance between capturing detailed features and maintaining computational efficiency.

**FIGURE 6:**
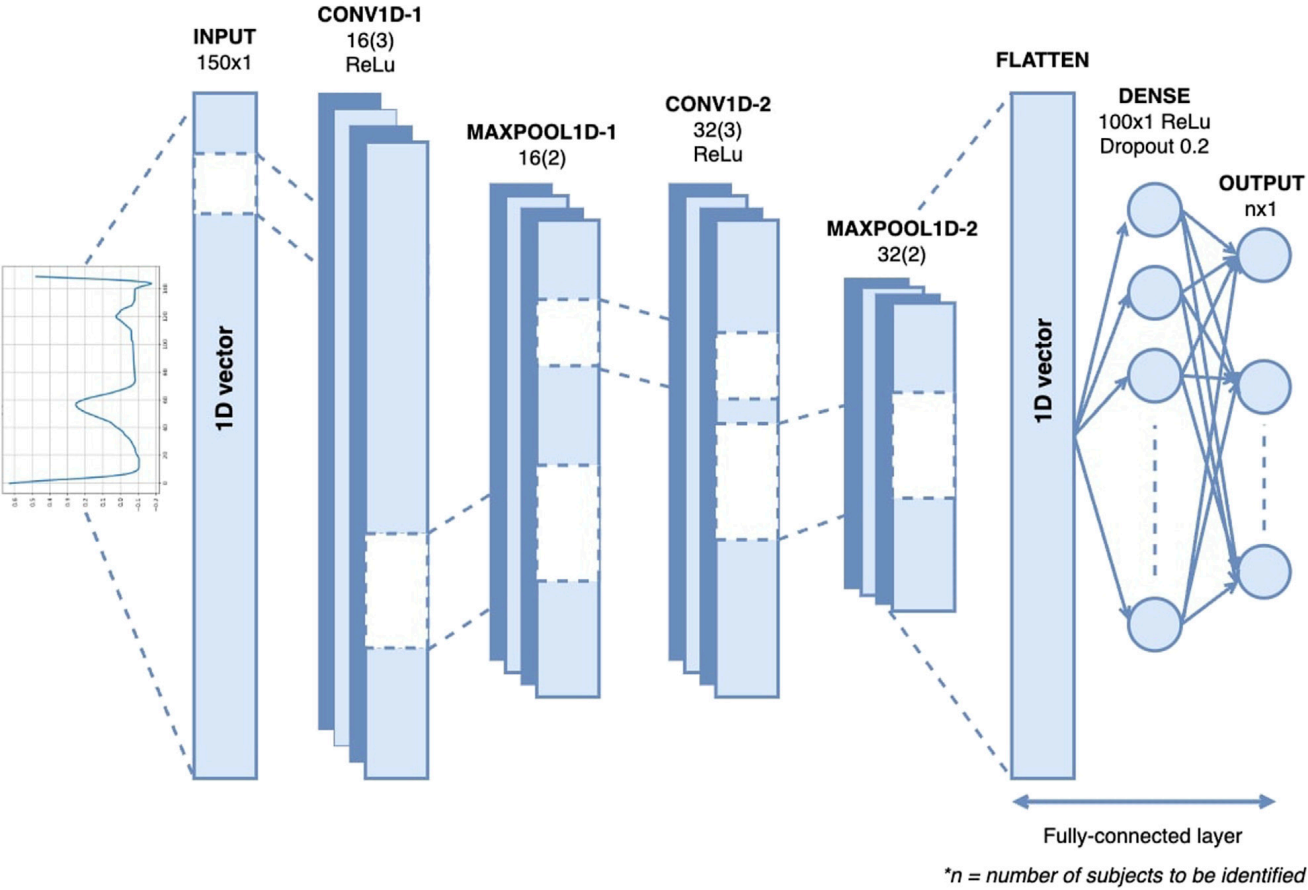
The architecture of 1D-CNN for single heartbeat ECG-based identification.

Figure 6 shows the architecture of the 1D-CNN used in this study. It is composed of two convolutional layers, two max-pooling layers, a flattened layer, one dense layer, one dropout layer, and a Softmax layer. The convolutional layer uses ReLU neurons to introduce non-linearity to the network, promoting the development of a more profound representation. The first and second convolutional layers consisted of 16 and 32 kernels, respectively, with the same filter size of 3. The max-pooling layer with a pool size of two is placed after each of the convolution layers to reduce the spatial dimensions of the matrices or the number of parameters while preserving the important features. The flattened layers transformed the output of the last max-pooling layer into a 1D vector. One dense layer, a fully connected layer with 100 neurons, and the ReLU activation function are added after the flattened layer to interpret the features extracted by the convolutional layers. Dropout regularization was applied at a rate of 0.2 to reduce overfitting by randomly setting a fraction of the input units to zero. The final layer of the network is a fully connected layer with a softmax activation function that serves as the classification function for the authentication task. The hyperparameters that we used in this study can be seen in Table 2.

**TABLE 2:** Hyper-parameter settings of the proposed model.

Parameter	Value
Learning Rate	0.001
Optimizer	Adam
Batch Size	32
Epoch	500
Loss	Categorical Cross-Entropy
Regulation/Validation	Early Stopping

### 2.4 Experimental settings

The overall experiment in this study was carried out using Keras 2.14.0 and Python 3.10.12 on the desktop computer with the specification of Windows 11 (64-bit), 65 GB RAM, AMD Ryzen 5 5600X, and NVIDIA GeForce RTX 3060 32 GB. We performed experiments using two scenarios: with imbalanced datasets and balanced one. For each scenario, we divided the datasets into a training set, validation set, and testing set with a ratio of 60:20:20, respectively.

#### 2.4.1 Scenario-1: Training with imbalanced datasets

In this scenario, we used imbalanced datasets, that is, the number of beat samples representing each subject varied. In this case, we applied k-fold cross-validation with *k* = 5 to ensure that every observation data in the datasets has a chance to appear in the training-validation set, reducing variability and overfitting. We then evaluated the performance of the proposed framework.

#### 2.4.2 Scenario-2: Training with balanced datasets

In this scenario, we intend to address the potential bias arising from the class imbalance present in our datasets, as the machine learning model tends to favor the majority classes during the learning process, resulting in poor performance on the minority class instances. To address this issue, we employed the SMOTE (Synthetic Minority Over-sampling Technique) algorithm developed by Chawla et al. (2002) for oversampling the minority class samples. This process involves.

1. Identify the minority class (the class with fewer instances) and the majority class (the class with more instances) in the dataset.
2. For each sample in the minority class, the algorithm finds its k-nearest neighbors from the same class. The value of k determines the number of nearest neighbors to consider.
3. For each minority class sample, the algorithm randomly selects one of its k-nearest neighbors. Then, it creates a new synthetic sample by interpolating between the selected minority sample and its nearest neighbor. This interpolation is performed by calculating the vector between the two samples and multiplying it by a random value between 0 and 1. The resulting vector is then added to the original minority sample, creating a new synthetic sample.
4. Repeated step 2 and 3 for all samples in the minority class, generating synthetic samples for each one.
5. Combine synthetic samples with the original dataset to achieve a balanced class distribution.

SMOTE has been widely adopted in the field of machine learning to mitigate the effects of class imbalance and improve the performance of classification models on minority class samples. And todate, many extensions and alternatives to the original SMOTE algorithm have been proposed, including Borderline-SMOTE (Han et al., 2005), Adaptive Synthetic Sampling (ADASYN) (He et al., 2008), and Density-Based Synthetic Minority Over-sampling Technique (DBSMOTE) (Bunkhumpornpat and Sinapiromsaran, 2009). In this work, we utilized the original SMOTE implementation from the imbalanced-learn library in Python (Lemaitre et al., 2017) with k = 5.

**FIGURE 7:**
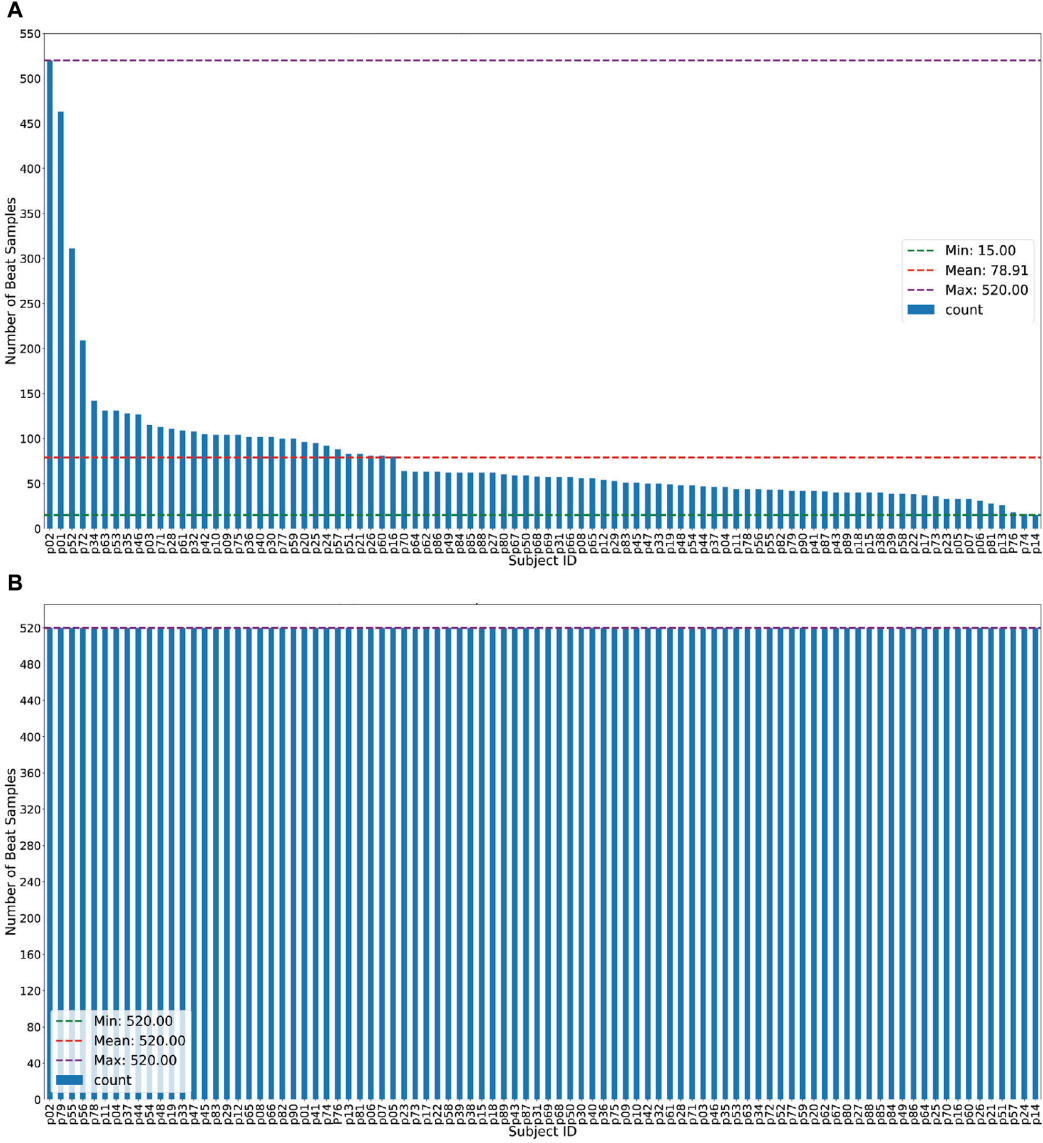
The illustration of samples size for each subject used in this study. **(A)** Example case of imbalanced samples size representing each subject on ECG-ID dataset. **(B)** Example case of balanced sample size per subject on ECG-ID dataset (after applying SMOTE Algorithm).

An illustration of this balancing procedure using SMOTE is presented in Figure 7. The number of samples for each subject was set to the same value: (a) equal to the mean value of the original sample distribution and (b) equal to the maximum value of the original sample. To generate synthetic instances of the minority class (i.e., the class with fewer instances), we interpolated the chosen instance with its k-nearest neighbors (*k* = 5). This is performed by choosing a random neighbor and computing the difference between the feature values of the chosen instance and the neighbor. The difference is then multiplied by a random value between 0 and 1 and added to the feature values of the chosen instance. This process was repeated until the ratio between the minority and majority classes was 1:1 (equal). Then, we trained using the same proposed framework and evaluated its performance.

### 2.5 Performance evaluation

The evaluation was performed on both the independent and mixed datasets. Since our problem is identification (a multi-class problem), we calculated the performance metrics using the following approach.

1. We first constructed a confusion matrix to summarize the performance of the classification model.
2. Overall accuracy was computed as the ratio of correctly classified instances (both true positives and true negatives instances) to the total number of instances.
3. We computed precision, recall, and F1-score for each class individually. These metrics help us understand the model’s performance in terms of correctly identifying each class (precision), capturing all relevant instances (recall), and balancing precision and recall (F1-score).
4. Those metrics were then macro-averaged to provide a single performance score across all classes. Macro-averaging treats each class equally by computing the metrics for each class independently and then taking the average, which is useful for understanding the performance across all classes without being biased towards more frequent classes.

The mathematical formulation of those metrics was defined as follows:

$$Accuracy=\frac{TP+TN}{TP+FP+TN+FN}$$ (5)

$$Precision=\frac{TP}{TP+FP}$$ (6)

$$Sensitivity=\frac{TP}{TP+FN}$$ (7)

$$F1-Score=2\times \frac{Precision\times Sensitivity}{Precision+Sensitivity}$$ (8)

where TP is a true positive, FP is a false positive, TN is a true negative, and FN is a false negative. TP refers to the number of test-set correctly identified as positive when they are positive. In this case, for example, if the test-set is subject-1, then the model correctly classifies it as subject-1. FP is otherwise. This reflects the number of test-set identified in the incorrect class. TN indicates the number of test sets correctly rejected by the model when they do not belong to the class. Meanwhile, FN indicates that the model fails to identify test sets as positive when they are actually positive.

Accuracy measures the proportion of correct predictions (both true positives and true negatives) among the total number of examined cases. In the context of an authentication system, this indicates how often the system correctly identifies or rejects an individual. Precision, also known as the positive predictive value, measures the proportion of positive identifications that are actually correct. High precision indicates that the system rarely misidentifies an individual. Sensitivity, also known as the Recall or True Positive Rate, measures the proportion of actual positives that are correctly identified. High sensitivity indicates that the system correctly identifies and authenticates legitimate individuals most of the time, thereby avoiding wrongly denied access. F1-score takes the harmonic mean between precision and sensitivity. It provides a single measure to evaluate the overall effectiveness of an authentication system when both false positives and false negatives are equally costly or undesirable.

## 3 Results

### 3.1 Performance of the proposed framework with imbalanced datasets

First, we observed the performance of our proposed framework on different ECG record databases with various numbers of subjects (participants) included in the study. The duration of the ECG records in each database varies, which in turn leads to an imbalanced number of samples for each subject. For example, in the ECG-ID database, subject-2 had up to 520 beat samples, while subject-14 had only 15 samples (see Figure 7A). To evaluate the performance of the system on such imbalanced datasets, we applied 5-fold cross-validation during the training process. Table 3 presents the classification accuracy of the proposed system across different databases, particularly with imbalanced datasets.

**TABLE 3:** The performance of the proposed framework with imbalanced datasets

No.	Database	# of Subjects	Distribution of samples size per subject			Accuracy (%)
			Min.	Mean	Max.	
1.	NSRDB	18	227	370	718	99.26
2.	MIT-ARR	27	71	197	429	98.82
3.	MIMIC-III	83	100	359	823	98.71
4.	ECG-ID	90	15	78	520	95.69
5.	MIXED-1^a^	173	15	213	823	98.44
6.	MIXED-2^b^	200	15	211	823	95.59
7.	MIXED-3^c^	218	15	224	823	93.38

^a^ MIXED-1: ECGID + MIMIC III;

^b^ MIXED-2: ECGID + MIMIC III + MIT-ARR;

^c^ MIXED-3: ECGID + MIMIC III + MIT-ARR + NSRDB.

The assessment of individual datasets showed that the NSRDB database with 18 subjects had a minimum of 227 beat samples per subject, a mean of 370, and a maximum of 718, resulting in an accuracy of 99.26%. The MIT-ARR database with 27 subjects had a sample distribution ranging from a minimum of 71 to a maximum of 429 per subject, with an average of 197. The accuracy of this database was 98.82%. MIMIC-III, a larger database with 83 subjects, in which each subject had between 100 and 823 samples, averaging 359 samples each, achieved an accuracy of 98.71%. The ECG-ID database, which included 90 subjects, had a range of 15–520 samples per subject and an average of 78, achieving an accuracy of 95.69%.

The assessment of the combined databases showed that the MIXED-1 dataset, comprising 173 subjects from ECG-ID and MIMIC-III, attained an accuracy of 98.44%. The number of samples per subject in this dataset ranged from 15 to 823, with a mean of 213. In the second combination, the MIXED-2 dataset, with 200 subjects, the sample distribution per subject was the same as MIXED-1, and the accuracy dropped to 95.59%. Finally, the MIXED-3 dataset, the largest dataset in this study, involving 218 subjects with the same range of samples as MIXED-1 and MIXED-2 but a slightly higher mean of 224, obtained an accuracy of 93.38%.

### 3.2 Performance of the proposed framework with balanced datasets

The proposed framework was then evaluated using balanced datasets. Balanced datasets were obtained by up-sampling and down-sampling the minority and majority classes, respectively, to equal values: (i) mean and (ii) the maximum value of the original sample distribution. Figure 7B) illustrates the appearance of balanced datasets. Table 4 presents the performance evaluation of the proposed framework when applied to the balanced datasets, detailing the number of subjects, number of samples used per subject, and performance metrics (accuracy, precision, sensitivity, and F1-score) achieved for each dataset.

**TABLE 4:** The Performance of the proposed framework with the balanced datasets.

No.	Database	# Subjects	# Samples per Subject	Acc. (%)	Prec. (%)	Sens. (%)	F1 (%)
1	NSRDB	18	370	100.00	100.00	100.00	100.00
			718	100.00	100.00	100.00	100.00
2.	MIT-ARR	27	197	100.00	100.00	100.00	100.00
			429	100.00	100.00	100.00	100.00
3.	MIMIC-III	83	359	98.95	98.89	98.93	98.88
			823	99.78	99.78	99.77	99.77
4.	ECG-ID	90	78	97.78	98.01	97.85	97.76
			520	99.88	99.88	99.88	99.88
5.	MIXED-1^a^	173	213	98.54	98.59	98.55	98.52
			823	99.74	99.75	99.76	99.75
6.	MIXED-2^b^	200	211	96.72	96.9	96.64	96.57
			823	99.7	99.72	99.7	99.71
7.	MIXED-3^c^	218	224	93.02	93.1	93.55	92.83
			823	99.7	99.79	99.67	99.66

^a^ MIXED-1: ECGID + MIMIC III;

^b^ MIXED-2: ECGID + MIMIC III + MIT-ARR;

^c^ MIXED-3: ECGID + MIMIC III + MIT-ARR + NSRDB.

The results demonstrated exceptional accuracy, with two datasets (NSRDB and MIT-ARR) achieving perfect scores across all metrics, indicating a 100% success rate in authenticating all subjects. These scores were achieved by balancing the number of beat samples per subject relative to either the mean or maximum value of the total sample distribution within the corresponding dataset. For the NSRDB, the number of beat samples per subject was set at 370 and 718, respectively. Whereas for MIT-ARR, it was set to 197 and 429, respectively.

For the MIMIC-III dataset with 83 subjects, the proposed method reached an accuracy of 98.95% when using 359 beats per subject, and 99.78% when using 823 beats per subject. The same upward trend was observed in other metrics, including precision, sensitivity, and F1-score when the number of samples per subject was increased.

For the ECG-ID, with a larger subject size, (i.e., 90 subjects), the authentication accuracy reached 97.78% with 78 samples per subject and increased to 99.88% with 520 samples per subject. Precision, sensitivity, and F1-score also increased with an increase in the number of samples per subject. In this case, the enhancement rate is approximately 2% for each metric.

The mixed datasets, which were designed to assess the framework under more varied conditions, slightly reduced the performance metrics; however, they remained impressively high. For the MIXED-1 dataset, the performance scores remained above 99%. On the MIXED-2 and MIXED-3 datasets, which are more diverse, all metric scores suffer at approximately 93%. Later, these scores could be elevated to above 99% after employing more samples per subject, that is, up to 823. As we can see, the MIXED-3 datasets, the broadest dataset, demonstrated the lowest scores among the datasets, with 99.7% accuracy, 99.79% precision, 99.67% sensitivity, and 99.66% F1-score.

### 3.3 Comparison with the state-of-the-art methods

We compared the results of our proposed framework with those of previous studies using the same single heartbeat input to the deep-learning network. In general, our proposed framework’s performance is comparable to or superior to state-of-the-art methods when evaluated on different datasets that have different characteristics and conditions, as shown in Table 5.

**TABLE 5:** The comparison of the proposed framework with the state-of-the-art methods using single heartbeat input.

References	Segment	Database name	#Of subjects	Acc (%)
El Boujnouni et al. (2022)	R-centered	NSRDB	18	100
Ihsanto et al. (2020)	R-centered	MIT-ARR	47	89.58
Prakash et al. (2023)	R-centered	ECG-ID	90	91
AlDuwaile and Islam (2021)	R-centered	ECG-ID	90	97.28
	R-R	PTB	100	98.0
Hong et al. (2019)	R-centered	PTB	200	97.84
Proposed framework	R-R	NSRDB	18	100.00
		MIT-ARR	27	100.00
		MIMIC-III	83	99.78
		ECG-ID	90	99.77
		MIXED-1^a^	173	99.74
		MIXED-2^b^	200	99.7
		MIXED-3^c^	218	99.7

^a^ MIXED-1: ECGID + MIMIC III.

^b^ MIXED-2: ECGID + MIMIC III + MIT-ARR.

^c^ MIXED-3: ECGID + MIMIC III + MIT-ARR + NSRDB.

Our proposed framework exhibited optimal performance when applied to a small number of subjects. A perfect accuracy rate of 100% consistent with the work of El Boujnouni et al. El Boujnouni et al. (2022) who used an R-centered segment to create a single heartbeat sample. The same accuracy rate of 100% was also obtained when authenticating 27 subjects with abnormal heart conditions on the MIT-ARR database. Using the same MIT-ARR database, the former work of Ihsanto et al. (2020) achieved 89.58% accuracy when using a single heartbeat input. However, they included more subjects (n = 47).

Attempting to use a larger subject population, our framework achieved a remarkable accuracy of 99.78% when authenticating 83 subjects on the MIMIC-III dataset. It slightly increase to 99.88% when applied to the ECG-ID dataset comprising 90 subjects. These results demonstrate the superior performance of our framework, beating the previous works that used a single heartbeat paradigm on the same ECG-ID dataset, that is, approximately 9% higher than (Prakash et al., 2023) and 2.5% higher than (AlDuwaile and Islam, 2021). Furthermore, AlDuwaile and Islam (2021) investigated the feasibility of using the R-R segment to authenticate 100 individuals on the PTB dataset and achieved 98% accuracy. Our proposed frameworks still showed a better accuracy (99.94%) when authenticating more individuals (173 subjects) but using a different dataset, namely, the MIXED-1 dataset, which was constructed from two different characteristic datasets.

Hong et al. (2019) used the PTB database consisting of 200 subjects to evaluate their proposed method and achieved an accuracy of 97.84%. Meanwhile, our proposed frameworks achieved 99.7% accuracy when authenticating the same number of subjects (200 subjects) but using different datasets. In this case, we used the MIXED-2 dataset, derived from a combination of ECG-ID, MIMIC-III, and MIT-ARR databases, which have different characteristics and conditions of ECG recordings. Moreover, using a larger number of subjects, that is, 218 on the MIXED-3 dataset, our proposed framework still surpasses the work of Hong et al. (2019), achieving 99.7% accuracy.

Furthermore, we compared our proposed framework using 1D-CNN with state-of-the-art methods that employ 2D-CNN for ECG-based identification task. The comparison is based on the total number of parameters in the CNN model and the Big O notation, which represents the computational complexity of the algorithm (see 6. The Big O notation provides the theoretical time and space complexities of algorithms or models as the input size grows, allowing the analysis of efficiency and resource requirements. The Big O notation is denoted using the mathematical symbol O, followed by an expression that represents the complexity. In this case, we used O(n) (Linear Time), indicating the running time grows linearly with the input size. From 6, we can observe that our proposed model using 1D-CNN has significantly fewer parameters (117,770 to 137,948) compared to the other methods that use 2D-CNN architectures (ranging from 23,800 to 2,664,546 parameters). The Big O complexity of the proposed 1D-CNN model is O (100n), which is lower than the complexities of the 2D-CNN models, such as O (1000000 + 2048n), O (256 h), O (1792n), and O (1536n).

## 4 Discussion

This study investigated the potential use of a single heartbeat taken from the R-R segment of ECG for human authentication by proposing a 1D-CNN framework comprising only two layers of convolution. Many studies have suggested the CNN framework using a short segment around R peak (R-centered paradigm) (Hong et al., 2019; Pinto and Cardoso, 2019; AlDuwaile and Islam, 2021; Srivastva et al., 2021; Prakash et al., 2023). We, on the other hand, investigated the authenticity of a segment between two consecutive R peaks with the point of view that it holds more holistic information of a single heartbeat instance because it encapsulates a complete systolic and diastolic phase. An ECG-authentication system with a single heartbeat input renders a challenge such as insufficient information to differentiate individuals and less adaptive to variations in the heart rate and rhythm within the same individual. The intra-individual variations stem from factors such as mental state, emotional condition, experimental setup or acquisition period, physical wellbeing, or drug consumption, as well as changes in lifestyle or individual traits (Melzi et al., 2023). Those variations were more noticeable in the signals recorded across separate sessions. Nevertheless, ECG has also been reported to be sufficiently stable over the years. Thus, choosing the appropriate segment to represent a single heartbeat is of utmost importance in this context because it can improve the learning process, authentication speed, and accuracy, leading to high acceptability in real-world scenarios.

The inconsistencies in the number of samples per subject across databases posed a challenge. Despite these obstacles, our proposed framework achieved impressive levels of accuracy: NSRDB, 99.26%; MIT-BIH Arrhythmia, 98.82%; MIMIC-III, 98.71%; and ECG-ID, 95.69% (3). Furthermore, the system’s ability to perform effectively on combined databases, such as MIXED-1, MIXED-2, and MIXED-3, with accuracies ranging from 93.38% to 98.44%, highlights its capacity to effectively manage data variability and distribution—an essential characteristic for real-world scenarios where data imbalance is prevalent. The investigation of imbalanced datasets is of great importance as it reflects real-world clinical scenarios in which data are often unevenly distributed. For instance, the highest accuracy obtained on the NSRDB dataset demonstrates the framework’s proficiency in handling moderate variations in the sample size per subject. This could indicate either more distinct ECG data per subject within this dataset or an optimal tuning of the system to its specific characteristics. However, a progressive decline in accuracy was observed when tested on larger, single-source, and mixed datasets. The highest accuracy was gained for the MIXED-1 dataset, while the lowest accuracy was gained for MIXED-3. This trend aligns with expectations because mixed datasets introduce greater variability by combining different data sources.

To address the skewed data distribution commonly found in real-world scenarios, we applied SMOTE algorithm (Chawla et al., 2002) from the imbalanced-learn Python library (Lemaitre et al., 2017) to achieve uniform sample distribution across individuals. Although numerous extensions and alternatives to the original SMOTE algorithm have emerged over the years, such as Borderline-SMOTE, Adaptive Synthetic Sampling (ADASYN), and Density-Based SMOTE (DBSMOTE), we decided to utilize the original SMOTE algorithm for two primary reasons. Firstly, the original SMOTE algorithm has undergone extensive research and broad adoption within the machine learning domain, making it a well-established and reliable technique for balancing the distribution of classes in imbalanced datasets. Secondly, the imbalanced-learn library provides a simple and robust implementation of SMOTE, ensuring consistent and reproducible results across different experiments and datasets. While more advanced variations may offer incremental improvements in specific scenarios, the original SMOTE already provided satisfactory results for our problem. The performance was improved significantly, achieving 100% scores across all metrics (accuracy, precision, sensitivity, and F1-score) on individual NSRDB and MIT-ARR datasets (see Table 4). This outstanding performance is further exemplified in the individual MIMIC-III and ECG-ID datasets, surpassing 99.7% for all performance metrics, indicating the exceptional competency of the framework in authenticating larger populations. The framework maintained high-performance metrics when tested on combined datasets, particularly on the MIXED-3 dataset, the broadest dataset comprising 218 subjects, achieving 99.7% accuracy and 99.79% precision. The results strongly suggest that employing a balanced approach to sample distribution significantly enhances the system performance, not only in single-source datasets but also in more diverse and complex mixed datasets. This outcome substantiates the adaptability and reliability of the proposed framework across a wide range of ECG databases.

In comparison with existing state-of-the-art methods, our framework that incorporates balanced sample distribution has shown superior performance in most cases, especially under the single heartbeat paradigm (see Table 5). This superiority is evident when matched against the best-reported accuracy on the NSRDB database (El Boujnouni et al., 2022), and significantly surpasses other methods on the MIT-ARR (Ihsanto et al., 2020) and ECG-ID databases (AlDuwaile and Islam, 2021; Prakash et al., 2023). When tested on a larger population with more than 100 subjects, our frameworks obtained 99.7% accuracy with combined datasets, surpassing the study of AlDuwaile and Islam (2021) and Hong et al. (2019) that used single-source PTB dataset. Furthermore, we quantified the benefits of using 1D-CNN over 2D-CNN model for single heartbeat ECG identification in terms of total parameters and Big O complexity as shown in Table 6. The lower number of parameters and computational complexity in the proposed 1D-CNN model can be attributed to the fact that it operates directly on the 1D ECG signal, avoiding the need for transformation to a 2D representation, as required by 2D-CNN models. The reduced number of parameters and lower Big O complexity suggest that the 1D-CNN model can be trained and deployed more efficiently, potentially leading to faster inference times and lower memory requirements. These benefits make the proposed approach attractive for real-time and resource-constrained applications in ECG-based identification systems.

**TABLE 6:** The complexity comparison of the proposed framework (using 1D-CNN) with the state-of-the-art methods (using 2D-CNN).

References	Deep learning model	Total parameters	Big O complexity
Hong et al. (2019)	2D-CNN (Inceptionv3)	2,459,546–2,664,546	O (1000000 + 2048n)
Donida Labati et al. (2019)	2D-CNN	891,090–941,490	O (256n)
AlDuwaile and Islam (2021)	2D-CNN	380,714–739,114	O (1792n)
El Boujnouni et al. (2022)	2D-CNN	23,800–243,560	O (1536n)
Proposed framework	1D-CNN	117,770–137,948	O (100n)

Although the proposed ECG-based authentication system demonstrates high performance across various datasets, several limitations must be acknowledged.

1. The datasets used for validating the framework involve ECG signals collected continuously over short periods. This controlled environment ensures consistency in the ECG signals but does not account for long-term variability. ECG signals can vary over time due to changes in the individual’s physiological and psychological state, health conditions, and environmental factors. Therefore, the system’s effectiveness in authenticating individuals based on ECG signals collected a week, a month, or a year later remains uncertain.
2. The current validation is a closed-set scenario, where the system knows all potential subjects during the training phase. This is a common assumption in biometric authentication but does not reflect real-world scenarios where new, previously unseen individuals may attempt authentication. In an open-set environment, the system must not only correctly authenticate known subjects but also accurately reject unknown subjects. The performance metrics presented do not account for this scenario, and the system’s ability to handle open-set conditions has not been tested.
3. The variation in sample size per subject significantly affects the system’s ability to learn and generalize, especially when the distribution is heavily skewed to certain subjects. This observation emphasizes the need for algorithmic improvements to enhance the ability of the framework to handle complex and diverse data.

Thus, future enhancements should focus on collecting and analyzing long-term ECG data to evaluate the system’s performance over extended periods. This would provide insights into the framework’s robustness and adaptability to temporal variations in ECG signals. The system’s performance also should be investigated in open-set scenarios by incorporating techniques such as thresholding, anomaly detection, additional biometric modalities integration or additional validation steps to improve the system’s discriminative power (distinguishing between known and unknown subjects). Evaluating and enhancing the system’s open-set performance is crucial for practical deployment. Moreover, refining the framework’s capabilities should also be considered, particularly in managing larger and more diverse datasets, to solidify its position as a transformative solution in the field of biometric authentication.

## 5 Conclusion

This study has investigated the efficacy of using only a single heartbeat (R-R segment) for reliable human authentication, addressing a significant challenge in the biometric domain. By leveraging only two layers of 1D-CNN that process R-R segments from the ECG signal, this study simplifies the complexity of the deep learning architecture while maintaining good performance of the system. Comprehensive evaluations using the NSRDB, MIT-ARR, MIMIC-III, and ECG-ID databases, have explicitly demonstrated that the proposed framework can achieve high levels of authentication accuracy, matching, and even surpassing state-of-the-art methodologies. A notable result is a perfect authentication score (100%) on the NSRDB and MIT-ARR database while maintaining high accuracy on larger mixed datasets, which consolidates the framework’s applicability to both small and large subject populations. Furthermore, the hypothesis that balanced sample sizes per subject could elevate authentication accuracy was confirmed, thereby underscoring the importance of uniform data distribution in enhancing the performance of biometric systems. The integration of a strict thresholding protocol in the beat clipping process is instrumental in minimizing noise, thereby reinforcing the robustness of the authentication system. The findings of this study demonstrated the potential of employing a single heartbeat for ECG-based authentication in practical scenarios. This approach not only streamlines the authentication process but also enhances the security and reliability of biometric systems. Future directions include optimization of the framework for real-time processing and the exploration of its scalability and effectiveness in diverse and larger populations. The success of this research paves the way for more secure, efficient, and user-friendly authentication systems in an increasingly digital and interconnected world.

## Data Availability

The original contributions presented in the study are included in the article/Supplementary material, further inquiries can be directed to the corresponding author.
